# Maternal and Fetal Pharmacokinetics of Oral Radiolabeled and Authentic Bisphenol A in the Rhesus Monkey

**DOI:** 10.1371/journal.pone.0165410

**Published:** 2016-12-08

**Authors:** Catherine A. VandeVoort, Roy R. Gerona, Frederick S. vom Saal, Alice F. Tarantal, Patricia A. Hunt, Anne Hillenweck, Daniel Zalko

**Affiliations:** 1 Department of Obstetrics and Gynecology, University of California, Davis, California, United States of America; 2 California National Primate Research Center, University of California, Davis, California, United States of America; 3 Program on Reproductive Health and the Environment, Department of Obstetrics, Gynecology and Reproductive Sciences, University of California, San Francisco, California, United States of America; 4 Division of Biological Sciences, University of Missouri, Columbia, Missouri, United States of America; 5 Departments of Pediatrics and Cell Biology and Human Anatomy, School of Medicine, University of California, Davis, California, United States of America; 6 School of Molecular Biosciences, Washington State University, Pullman, Washington, United States of America; 7 Toxalim, Université de Toulouse, INRA (Institut National de la Recherche Agronomique), Toulouse, France; Universidad Miguel Hernandez de Elche, SPAIN

## Abstract

The present study was conducted in pregnant rhesus monkeys to determine the rapidity and extent to which BPA reaches the fetal compartment following oral ingestion, and the 24-hr fate of BPA. To assess metabolism changes during the course of pregnancy, we compared BPA biotransformation during the second and third trimesters in the same animals, measuring the levels of sulfated, gluronidated, and free BPA in maternal serum, amniotic fluid, and fetal serum. All animals showed measurable unconjugated and conjugated BPA in the fetal compartment and slow clearance compared to maternal serum. There were higher levels of BPA-G in amniotic fluid at 150 days gestation compared to 100 days gestation, as well as higher levels of BPA-G than BPA-S. We also monitored ^3^H-BPA (and metabolites) in key tissues and excreta from a mother and fetus and from a non-pregnant female. The elimination of radioactivity was rapid, but residues were still detectable 24 hr after dosing in all tissues analyzed. These data suggest that, in primates, rapid maternal processing of BPA does not alleviate the risk of exposure to the developing fetus. This study elevates concerns about levels of current BPA human exposure from potentially a large number of unknown sources and the risks posed to developing fetuses.

## Introduction

The use of bisphenol A (BPA) in a wide range of consumer products brings humans into daily contact with this chemical, and data from biomonitoring studies suggest that BPA and its metabolites can be detected in nearly all human subjects [1]. Indeed, epidemiological data suggest that, in the US, exposure to BPA is nearly constant [2]. Despite the pervasiveness of exposure, our understanding of the levels and routes by which we are exposed and how our bodies metabolize BPA remains woefully inadequate [3].

It was assumed for many years that the major route of human exposure to BPA is through the ingestion of contaminated food and beverages. Data from numerous biomonitoring studies, however, have reported far higher levels of unconjugated BPA in serum than expected based on the assumption that BPA is absorbed from the gastrointestinal tract [3–4], and growing evidence suggests that non-oral routes contribute significantly to human BPA exposure [2–3,5–7]. This is important, because it means that inferences about the levels and duration of human exposure to unconjugated (bioactive) BPA that have been based on the assumption that BPA is rapidly conjugated and inactivated significantly underestimate human exposure.

In the rodent, dog and monkey, intra-gastric gavage results in relatively rapid conjugation of BPA and bioavailability of less than 1% [8–10]. In monkeys, however, BPA delivered in a piece of fruit resulted in BPA bioavailability of ~7%, indicating higher than expected unconjugated BPA in serum relative to bioavailability after intra-gastric gavage. This difference was attributed to the monkeys slowly chewing the fruit, and some monkeys not swallowing all of the fruit for an extended period of time [7,11].

Data from direct studies in human adults also provide evidence that the route of exposure influences the levels and duration of exposure to bioactive BPA. The first metabolism study in humans involved a single dose administered in a capsule [12]. Although the assay was insufficiently sensitive for measurement of the unconjugated compound, levels of conjugated BPA led authors to conclude that BPA is rapidly conjugated and excreted in humans. This is consistent with data from experimental studies, since capsule delivery, like gavage, should result in low bioavailability BPA in serum. In contrast, as in monkeys fed BPA in a piece of fruit [11], the delivery of BPA in a cookie resulted in higher BPA bioavailability in humans [13]. Interestingly, however, a recent study administering BPA in soup led to low bioavailability [14], suggesting that the rapidity with which the chemical is swallowed influences exposure. This is consistent with the finding that residence time in the mouth is a critical variable that affects bioavailability in dogs [9], and suggests that buccal/sublingual absorption attenuates the rapid “first-pass” metabolism of gavage-administered BPA.

A rapid increase in unconjugated BPA in serum also has been reported from transdermal absorption from handling thermal receipt paper. Importantly, levels of bioactive BPA were sustained, with higher bioactive than conjugated levels persisting in serum for over 90 min following exposure [6]. Thus, based on the available data from human and experimental studies, entry of BPA into the body by sublingual, transdermal or respiratory exposure should lead to higher and sustained levels of bioactive BPA in serum than predicted on the basis of intra-gastric gavage.

This is sobering, given that the low levels induced by oral BPA exposure have been reported to significantly impact the fetus, altering the development of a wide range of organ systems, including brain, reproductive tract, lung, and mammary and prostate glands, and leading to behavioral changes, reduced fertility, metabolic disorders and cancer in adult laboratory animals [15–17]. Although most studies reporting effects have used rodents, recent studies in the rhesus monkey provide evidence of a similar wide range of effects in response to blood levels of unconjugated BPA below those reported in human biomonitoring studies [3,11,18–23].

Concerns about interspecies differences in the metabolic fate of BPA in mice and primates [24] have made the rhesus monkey a critical model in understanding the pharmacokinetics of BPA. BPA readily crosses from maternal blood into the fetal compartment in the monkey [7,10], and a single bolus intravenous (IV) dose has been reported to produce measurable levels of unconjugated BPA in fetal blood in less than an hour [10]. Further, a 50-day exposure using daily oral dosing or continuous exposure via implanted Silastic capsules resulted in detectable unconjugated BPA in fetal serum and demonstrated that BPA pharmacokinetics are altered by pregnancy and change over the course of gestation [7].

Assessing the risk posed by fetal exposure is not only complicated by metabolic differences due to exposure route and stage of gestation, but also by new concerns about BPA metabolites. The two major metabolites, BPA-monosulfate (BPA-S) and BPA-glucuronide (BPA-G) have been widely assumed to be both stable and to have no bioactivity. Further, the stability of these metabolites in the body is unclear: Like estrogen, which undergoes sulfation and desulfation, particularly during pregnancy [25] sulfated BPA could act as a “reserve”, yielding bioactive BPA following hydrolysis. In addition, many tissues contain ß-glucuronidases that can deconjugate glucuronidated metabolites [26–27], and deconjugation of BPA-G by ß-glucuronidase has been reported in the rat caecum; enterohepatic circulation of BPA in rodents is well established [28].

The present study was conducted in the pregnant rhesus monkey to determine the rapidity and extent to which BPA reaches the fetal compartment following oral ingestion, and the 24-hr fate of BPA in the pregnant female. To assess metabolism changes during the course of pregnancy, we compared BPA biotransformation during the second and third trimesters in the same animal, measuring the levels of sulfated, gluronidated, and free BPA in maternal serum, amniotic fluid, and fetal serum. We also monitored ^3^H-BPA (and metabolites) in key tissues and excreta from the mother and fetus. Our data suggest that, in primates, rapid maternal processing of BPA—even when it is orally ingested—does not alleviate the risk of exposure to the developing fetus.

## Materials and Methods

Monkeys. Five adult female rhesus macaques (*Macaca mulatta*) were housed at the California National Primate Research Center (CNPRC). Animals were caged individually with a 0600–1800 hours light cycle and at a temperature maintained between 25–27°C. Cage size depends on animal weight. Animals weighing less than 10kg were in a 4.3 square foot cage measuring 24 (L) x 27 (D) x 32 (H). Animals that were between 10kg and 15 kg were housed in a 6.4 square foot cage measuring 34 x 27 x 32. No animals exceeded 15 kg. Animals were fed a diet of Purina Monkey Chow and water ad libitum. Seasonal produce, seeds, and cereal were offered as supplements for environmental enrichment. Cages were made of stainless steel, and water was delivered to each cage by rigid PVC pipes and a “lixit” device. A wide array of enrichment is available at CNPRC including manipulation, food and visual/auditory activities. All food items were fresh and cage enrichment devices were determined to not be made of BPA-containing plastic to minimize any possible BPA exposure other than treatments. Only females with a history of normal menstrual cycles were selected for this study. Females ranged in age from 6 to 13 years. Four females were time-mated according to standard CNPRC procedures. Pregnancy was detected by ultrasound and an estimated day of conception (day 0) was assigned at the time of pregnancy detection based on the timed mating schedule [29]. At approximately 40 days gestation the sex of the conceptus was determined using established protocols and those dams with female fetuses were selected for the study (N = 4). Animal protocols were reviewed and approved by the Institutional Animal Care and Use Committee at the University of California, Davis prior to study initiation; all studies were conducted in accordance with the U.S. National Institutes of Health Guide for the Care and Use of Laboratory Animals.

## BPA Dosing and Sample Collection

### Pharmacokinetic study

Oral non-labeled BPA (400 μg/kg) was given daily to three pregnant females from approximately 100 to 150 days gestation (term ~165±10 days). The BPA dose was dissolved in a small (100–150 μL) volume of ethanol and administered in a piece of dried fruit. On days of blood and amniotic fluid sample collection BPA was administered via nasogastric intubation to prevent a possible delay in absorption due to cheek pouch food storing by some monkeys [7,11].

On sample collection days, maternal blood, amniotic fluid, and fetal blood were collected from Telazol anesthetized animals in sequence; maternal blood was obtained from a peripheral vessel and amniotic fluid and fetal blood (cardiocentesis) were collected under ultrasound-guidance using well-established methods [29]. Pre-treatment baseline values were obtained at approximately 95 days gestation (second trimester) and prior to the first maternal dose of BPA. At approximately 100 days gestation daily BPA dosing commenced and, following administration of the first dose, maternal and fetal samples were collected at 1, 2, and 4 hr post-administration. Animals were returned to housing and monitored by colony staff carefully after collection procedures. Near term (approximately 150 days gestation), the final BPA dose was administered, dams were anesthetized and samples were collected at 1, 2, and 4 hrs post-administration, with the final collection coordinated with the surgical removal of the gestational sac for fetal tissue collection. For gestational sac removal, animals were first anesthetized with Telazol followed by inhalation anesthesia during surgery, according to established CNPRC procedures. All dams were returned to the colony post-hysterotomy, monitored by colony personnel and administered analgesics as necessary.

### ^3^H-BPA metabolic balance study

Nasogastric intubation was used for two additional adult females (one pregnant and one non-pregnant) who received a single oral dose of ^3^H-BPA, corresponding to 2.50 MBq (6.76 x 10–11 mCi or 7.78 μg BPA/kg BW) and 1.42 MBq (3.84 x 10–11 mCi or 4.45 μg BPA/kg BW) for the pregnant (approximately 140 days gestation) and the non-pregnant female, respectively. For these animals, urine and feces were collected over a 24-hr period. Fluids, excreta, and tissues were recovered and stored at ≤-20°C until radioactivity quantification. Cages were washed with ethanol for radioactivity measurement and inclusion in the metabolic balance. Collected samples were sent to the INRA Research Centre in Food Toxicology (TOXALIM, Toulouse, France) for radioactivity quantification, extraction, and Radio-HPLC analyses in compliance with international regulations.

## Chemical Analysis

### Chemicals

Radiolabeled bisphenol A ([^3^H]-bisphenol A, radiochemical purity 99.9% checked by radio-High Performance Liquid Chromatography coupled to radioactivity detection (Radio-HPLC); specific activity 299.7 GBq/mmol) was purchased from Moravek Biochemicals and Radiochemicals (Brea, ST, USA). Monophase S, Flo-Scint II and Ultima Gold liquid scintillation cocktails were obtained from Perkin Elmer Life Sciences (Courtabœuf, France). All solvents were of analytical grade and were purchased from Fisher Scientific (Illkrich, France). Ultrapure water from Milli-Q system (Millipore, Saint Quentin en Yvelines, France) was used for HPLC mobile phases.

Reference standards for BPA and BPA-d16 were purchased from Sigma (St Louis, MO) while analytical grade standards for BPA sulfate, BPA glucuronide and ^13^C-BPA glucuronide were provided by the National Toxicology Program. BPA-free water was purchased from Aqua Solutions (Deer Park, TX) and analytical grade methanol and acetonitrile were obtained from Honeywell Burdick and Jackson (Muskegon, MI). Stock solutions of standards and internal standards were prepared at 1 mg/mL, aliquoted to 1 mL portions in amber vials and stored at ≤-80°C. All calibration standards, ranging in concentration from 0.1 to 80 ng/mL, were prepared from the stock solution by serial dilution in the appropriate matrix blank.

### Pharmacokinetic studies

Analysis of BPA, BPA-G and BPA-S in amniotic fluid and serum was performed by liquid chromatography-tandem mass spectrometry (LC-MSMS) as previously reported for serum [30]. Using Agilent LC 1260- AB Sciex 5500, all three analytes were measured simultaneously using electrospray ionization in negative polarity. Each analyte was monitored by multiple reaction monitoring using two transitions, and BPA-d16 and ^13^C_12_-BPA mono-β-D-glucuronide as internal standards: BPA- 227.0–133.1, 227.0–212.1; BPA glucuronide- 402.9–112.9, 402.9–226.9; BPA mono-sulfate-306.9–227.0, 306.9–212.1; BPA-d16- 241.0–142.2, 241.0–222.1; and ^13^C_12_-BPA mono-β-D-glucuronide- 415.0–112.8, 415.0–239.1.

Amniotic fluid and serum samples were thawed and centrifuged at 1811 x g for 10 minutes prior to their preparation for LC-MS/MS analysis by protein precipitation. Protein precipitation was done by adding 750 μL 95:5 (v/v) acetonitrile: methanol to 250 μL amniotic fluid. The resulting mixture was spun at 1811 x g for 5 minutes. The supernatant obtained after centrifugation was then gently evaporated under a stream of nitrogen gas, after which it was reconstituted in 10% methanol for column injection.

A 25-uL aliquot of the extract was used for each replicate injection of the sample. An Agilent Zorbax Extend-C18 column (4.6 x 100 mm, 1.8 μm) maintained at 50°C was used for chromatography. Chromatographic separation of the analytes was achieved by gradient elution using BPA-free water with 0.05% ammonium acetate (pH = 7.8) as mobile phase A and methanol with 0.05% ammonium acetate (pH = 7.8) as mobile phase B. The elution gradient employed was- 0–0.5 min = 30%B; 1 min = 75%B; 4 min = 100; 4–6 min = 100%B; and 6.01–12 min = 30% B. The method's limit of detection (LOD) for BPA and BPA glucuronide was 0.05 ng/mL while the method's LOD for BPA mono-sulfate was 0.025 ng/mL. Quantitation of each analyte was done by isotope dilution method using a 10-point calibration curve. Each analyte has a limit of quantitation (LOQ) of 0.1 ng/mL Method precision and recovery were validated for the assay and are reported in Tables 1 and 2. Method validation information has been published [30]. Pretreatment samples of maternal blood, fetal blood and amniotic fluid for each animal had no measurable BPA, thus verifying that sample contamination had not occurred. The issue of sample contamination with chemicals, which are ubiquitous in the environment, is not unique to BPA, and those experienced in developing assays are well aware of the required controls to ensure the absence of contamination [3].

**Table 1:** Validated method precision established for the direct analysis of BPA and its primary metabolic conjugates in amniotic fluid using LC-MS/MS.

Analyte	Precision (% CV)					
	Within Run (n = 5)			Between Run (n = 15)		
	Low 0.5 ng/mL	Med 5 ng/mL	High 20 ng/mL	Low 0.5 ng/mL	Med 5 ng/mL	High 20 ng/mL
**BPA**	4.0	2.5	1.5	6.0	5.5	4.8
**BPA-glucuronide**	4.5	2.5	2.8	8.5	7.8	8.2
**BPA-sulfate**	2.5	2.3	1.8	5.5	5.2	5.0

**Table 2:** Validated method recoveries established for the direct analysis of BPA and its primary metabolic conjugates in amniotic fluid using LC-MS/MS.

Analyte	Recovery (%)					
	Within Run (n = 5)			Between Run (n = 15)		
	Low 0.5 ng/mL	Med 5 ng/mL	High 20 ng/mL	Low 0.5 ng/mL	Med 5 ng/mL	High 20 ng/mL
**BPA**	90.5	94.5	94.5	88.0	94.5	93.0
**BPA-glucuronide**	87.5	90.4	92.0	84.5	89.0	90.2
**BPA-sulfate**	88.0	92.5	96.5	86.5	90.5	91.5

### Radioactivity quantification

Liquid samples: radioactivity contained in plasma, urine, serum, bile, and amniotic fluid was quantified by direct counting on a Packard scintillation counter (Model Tricarb 2100; Perkin Elmer, Courtabœuf, France) using Packard Ultima Gold (Perkin Elmer) as the scintillation cocktail. An external standard method was used for automatic quench correction. Tissues and organs: tissues were homogenized by dilaceration before sampling for radioactivity quantification. Aliquots of 0.2 to 0.5 g of wet tissue were combusted in a Perkin Elmer oxidizer (Model 307) using 15 mL Monophase S (Perkin Elmer) as scintillation cocktail. Three replicates were analyzed for each sample.

### Radio-HPLC analyses

Samples were analyzed on an Ultimate 3000 HPLC system (ThermoFisher, Villebon sur Yvette, France) connected for radioactivity detection to a radiometric flow scintillation analyzer Flo-one A500 (Perkin Elmer). FloScint II was used as scintillation cocktail. Metabolite separations were performed on a Zorbax SB-C18 column (5 μm, 250 x 4.6 mm, Agilent Technologies, Les Ulis, France) protected by a Kromasil C_18_ precolumn (5 μm, 10 x 4.6 mm). The mobile phases were ammonium acetate buffer 20 mM, pH 3.5/acetonitrile: 95%/5% in (A) and 10%/90% in (B), respectively. Solvents were delivered at a flow rate of 1 mL/min as follows: 0 to 4 min 100% A; 4 to 6 min linear gradient from 100% A to 15% B, 6 to 16 min 15% B; 16 to 18 min linear gradient from 15% B to 25% B, 18 to 28 min 25% B; 28 to 30 min linear gradient from 25% B to 30% B, 30 to 37 min 30% B; 37 to 39 min linear gradient from 30% B to 70% B; 39 to 50 min 70% B; 50 to 52 min linear gradient from 70% B to 100% B; 52 to 60 min 100% B. In such conditions, BPA’s retention time (R_*T*_) was 43 min. Metabolites were quantified by integrating the area under detected radioactive peaks.

### Enzymatic hydrolyses and metabolites identification

Given the low BPA doses used and the high specific radioactivity of ^3^H-BPA, direct MS characterization was not possible. Consequently, structural elucidation was carried out using biochemical tools and confirmed by co-elution with authentic standards synthesized as previously described by us [24,31]. ß-glucuronidase from *E*. *coli* and sulfatase from *A*. *aerogenes* (Sigma-Aldrich, Saint Quentin Fallavier, France) were used to hydrolyze conjugated metabolites in order to assess the formation of the BPA-G and BPA-S, respectively. ß-glucuronidase: enzyme hydrolysis was conducted by incubating samples in 1 mL sodium acetate buffer (200 mM, pH 5) containing 500 International Units of *E*. *coli* ß glucuronidase, as specified by the supplier. Sulfatase: samples were incubated with 1 mL Tris buffer (10 mM, pH 7.1) containing 1 unit of *A*. *aerogenes* sulfatase as specified by the supplier. ß-glucuronidase and sulfatase incubations were performed over 3 hr at 37°C in a shaking water bath. Blank incubations were performed without enzyme under the same conditions. At the end of the incubation period, proteins were precipitated with 3 mL of MeOH, the supernatant was evaporated under a stream of nitrogen, and the residue was solubilized in mobile phase A for HPLC analysis.

### Statistical analysis

Comparison between values was achieved using the Student t-test.

## Results

### BPA, BPA-G and BPA-S in maternal and fetal serum at 100 and 150 days gestation

**Fig 1:**
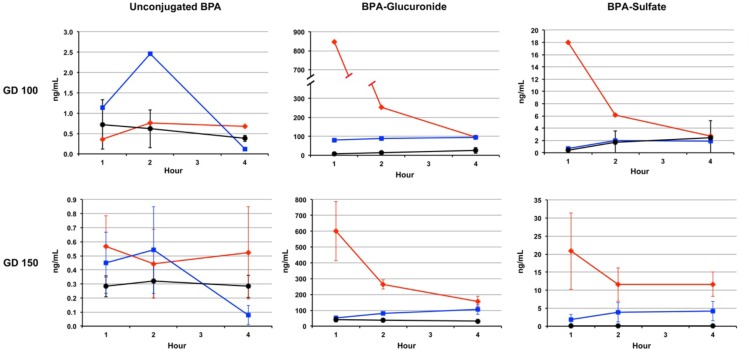
Levels of unconjugated BPA, BPA-G and BPA-S in maternal (red line) and fetal (blue) serum and amniotic fluid (black) at 100 (top panel, N = 2) and 150 days gestation (bottom panel, N = 3) following a single oral dose of BPA.

Levels of unconjugated BPA (Fig 1) were generally within a similar range in maternal and fetal serum at both gestational time points assessed, with the exception of one monkey at gestation day 100 that showed a spike in fetal serum unconjugated BPA 2 hours after maternal gavage, that resulted in a 20-fold higher level in fetal serum compared to maternal serum. In addition, samples from one monkey at the 100 day time point were damaged and thus, data from that animal is not include; thus, standard errors were not calculated for Day 100.

Although levels of unconjugated BPA were generally similar in maternal serum and fetal serum at gestation days 100 and 150 (Fig 1), differences in the levels of conjugates were evident. Maternal serum BPA-G and BPA-S were highest 1 hr after dose delivery and declined over the 4 hr assessment period. In contrast, levels of conjugated BPA in fetal serum tended to slightly increase between 1 and 4 hr after maternal dosing. Maternal and fetal serum levels of BPA-G were generally in the range of 100-fold higher than levels of unconjugated BPA, with highest ratios of glucuronidated to unconjugated observed at the 1 hr time point in maternal serum and the 4 hr time point in fetal serum. The levels of BPA-S were 10–30 times lower than BPA-glucuronide at the time points evaluated, but exhibited the same pattern as BPA-G, continuing to rise slightly through the 4 hr in fetal serum, while maternal serum levels declined. It is important to note that maternal levels of both BPA-G and BPA-S were higher than fetal levels at 1 and 2 hr after exposure, but not at the 4 hr time point.

The maternal and fetal serum and amniotic fluid levels of unconjugated BPA at 100 and 150 days gestation are shown in Fig 1. With the exception of one animal at 150 days, amniotic fluid levels of unconjugated BPA remained relatively stable between 1 and 4 hr after maternal dosing. There were higher levels of BPA-G at 150 days gestation compared to 100 days gestation, as well as higher levels of BPA-G than BPA-S. Amniotic fluid had a range of unconjugated BPA of 0.15 to 1.71 ng/mL, BPA-G of 3.56 to 65.54 ng/mL and BPA-S of 0.01 to 6.66 ng/mL. The pre-treatment collections of all samples at 100 days gestation were below the limits of detection for unconjugated BPA, BPA-G and BPA-S.

### Disposition of BPA over 24 hr after oral administration of ^3^H-BPA

**Fig 2:**
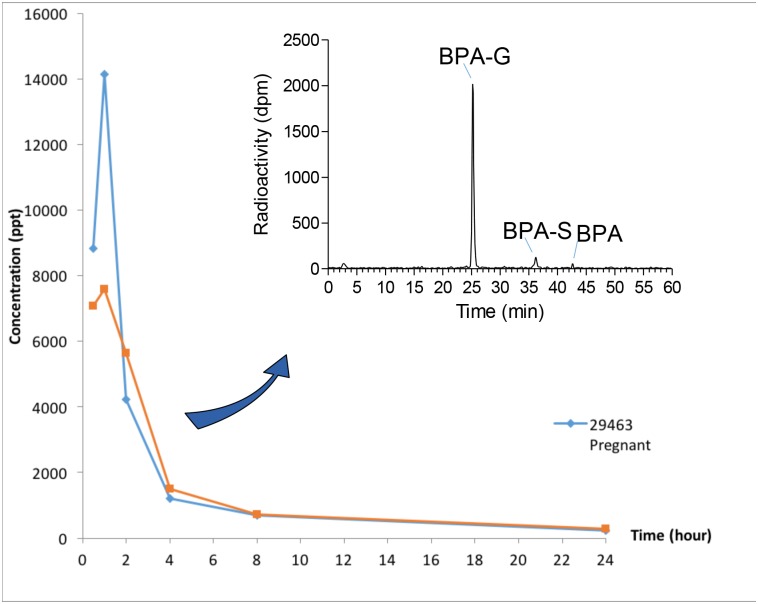
0–24 hr serum levels of BPA residues measured in a pregnant (blue) and a non pregnant (orange) Rhesus macaque dosed with 7.78 μg/kg and 4.45 μg/kg ^3^H-BPA, respectively. Results are expressed in pg/g (ppt) of BPA equivalents. A typical radio-HPLC profile corresponding to the analysis of serum radioactivity is displayed at the top right of the figure.

An early peak of ^3^H in serum (T = 1 hr) was observed in both the pregnant and the non-pregnant monkey dosed with ^3^H-BPA (Fig 2). The elimination of radioactivity was rapid, but residues were still detectable 24 hr after dosing [~220 and 286 parts per trillion (ppt; pg/g) in the pregnant and non-pregnant animal, respectively]. Direct radio-HPLC profiling could be carried out in both animals up to 4 hr after dosing (Fig 2 & Table 3), confirming the presence of a major peak identified as BPA-G based on retention time (Rt) comparison with the authentic standard, as well as specific biochemical hydrolyses, followed by R-HPLC controls. Likewise, we confirmed the presence of both the sulfate conjugate and parent compound in serum, although both BPA-S and BPA accounted only for a minor part of the detected radioactivity (Table 3).

**Table 3:** Respective radio-HPLC percentages of BPA glucuronide (BPA-G), BPA sulfate (BPA-S), unconjugated BPA, and a double glucuronide/sulfate conjugate of BPA (G-BPA-S, bile only) in monkey serum, bile, and urine samples. A = non pregnant animal. B = pregnant animal.

Sample	Animal	Time (hr)	BPA-G	BPA-S	BPA	G-BPA-S	Total
Serum	A	0.5	87.7	4.5	2.1		94.3
		1	89.2	3.8	3.3		96.3
		2	84.2	2.6	5.7		92.5
		4	87.3	4.8	2.5		94.6
Serum	B	0.5	94.5	3	0.9		98.4
		1	96.7	1.2	0.5		98.4
		2	86	3.8	1.5		91.3
		4	84.8	4.2	2.1		91.1
Bile	A	24	58.4	10.1	12.5	10.5	91.5
	B	24	61.8	8.1	7.5	12.2	89.6
Urine	A	0–24	87.4	0.39	4.79		92.6
	B	0–24	91.9	0.49	3.39		95.8
	Fetus (B)	24	87.2	0	0.97		88.17

In both ^3^H-dosed animals most of the radioactivity was excreted in urine, while fecal excretion was extremely limited (Table 4). Additional radioactivity (12–13% of the ^3^H dose) was recovered in cages, likely corresponding to urine that dried in cage pans. Despite the limited fecal excretion, roughly 2% of the administered radioactivity remained in the gastrointestinal tract of both animals 24 hr post-dosing, and nearly as much in bile (Table 4), corresponding to high biliary residue concentrations of 146 and 277 ppb (parts per billion) for the non-pregnant and the pregnant animal, respectively. In urine as well as in serum samples, only 2 metabolites were detected in addition to the parent compound. BPA-S and parent (unconjugated) BPA were present, but BPA-G was by far the predominant peak in urine (Table 3). Higher proportions of both BPA and BPA-S were found in bile samples, where an additional doubly glucuronide/sulfate conjugate of BPA was characterized through selective enzymatic hydrolyses and subsequent R-HPLC analyses.

**Table 4:** ^3^H-BPA 24 hr metabolic balance in a non-pregnant (A) and a pregnant (B) monkey orally dosed with 4.5 and 7.8 μg/kg BW ^3^H-BPA, respectively. Results are expressed in percentage of the administered dose.

	Non-Pregnant (A)	Pregnant (B)
Urine	83.05	90.64
GI Tract	1.58	2.88
Bile	1.83	1.93
Feces	0.11	0.001
Tissues (sum of)	0.23	0.046
Placenta		0.009
Fetus		0.001
SAmniotic fluid	N.A	0.025
TOTAL	86.79	95.53
Cages	12.33	13.12

Table 5 summarizes levels of ^3^H residues in tissues at 24 hr, for the non-pregnant animal and both the dam and fetus. BPA residues could be quantified in all tissues, and ranged from 10 to 100 ppt. Higher levels were measured in the kidney and the liver, as well as in the uterus and ovaries in both the pregnant and non-pregnant female. For the pregnant monkey, the overall transfer of BPA residues at 24 hours to the fetus was less than 0.03% of the dose, 5/6th of which was located in the amniotic fluid, and an additional 0.01% in the placenta. However, radioactivity at 24 hours could be detected in all fetal tissues and fluids, ranging from 22.5 ppt (brain) to 136.7 ppt (lung). The level of ^3^H residues measured in fetal serum was 119 ppt. Only BPA-G and parent BPA were detected in fetal urine collected from the bladder and amniotic fluid. In fetal urine, BPA-G was by far the predominant metabolite, and only traces of parent BPA (1%) were detected (Table 3). Conversely, although BPA-G was the major residue in amniotic fluid (182 ppt), a greater contribution of parent BPA (24.5 ppt) was observed in this sample.

**Table 5:** ^3^H residue levels in adult and fetal tissues and fluids, at 24 hr, in an adult female Rhesus macaque (A) orally dosed with 4.5 μg/kg BW ^3^H-BPA, and in a gravid dam (B) orally dosed with 7.8 μg/kg BW ^3^H-BPA, and the respective fetus. Results are expressed in ppt (ng/g) of BPA equivalents. Percent (%) of dose is the percentage of the administered dose.

Animal/Dose	Animal A 4.45 μg/kg		Animal B 7.77 μg/kg			
			Dam		Fetus	
	% of Dose	ppt	% of Dose	ppt	% of Dose	ppt
Liver	0.167	396.7	0.0302	146.5	0.00081	59.8
Kidney	0.031	405.1	0.0124	216.2	0.00031	104.6
Uterus	0.014	265.3	0.0287	165.2	0.00002	78.7
Ovaries	0.0014	595.8	0.0004	750.3	0.00001	84.3
Brain	0.0047	21.6	0.0019	13.2	0.00141	22.5
Heart	0.0022	20.6	0.0006	13.2	0.0001	59.9
Lung	0.0009	43.8	0.0018	29.5	0.00171	136.7
Adrenals	0.0008	97.7	0.0001	58.0	0.00001	60.7
Spleen		63.8	0.0004	47.3	0.00003	42.3
Skin		48.2		65.6		96.3
Muscle		49.8		28.3		50.1
Fat (Abdominal)		27.5		14.6		74.7
Fat (Cutaneous)		11.7		15.1		

## Discussion

Data from numerous human biomonitoring studies suggest far higher levels of serum unconjugated BPA than expected on the basis of experimental studies using gavage administration [32]. Further, while there is a considerable information on the pharmacokinetics of BPA in rodents, information on humans are very limited [3,33]. Studies comparing BPA pharmacokinetics in pregnant and non-pregnant women are not possible, as administration of BPA during pregnancy would not be ethically acceptable given the large amount of data showing a wide range of impacts to developing fetuses [15–17,30,34]. Given the importance of understanding human exposure levels, routes of exposure, and how our bodies metabolize BPA—especially during pregnancy—we turned to a nonhuman primate model, the rhesus monkey. The data presented here examine the levels of BPA and both major conjugates, BPA-glucuronide (BPA-G) and BPA sulfate (BPA-S) in maternal and fetal compartments during mid- and late gestation after gavage administration. Importantly, our data provide the most accurate assessment of BPA levels to date, since the LC-MSMS assay used to measure BPA has been shown to be accurate and contamination free in a NIH-sponsored round-robin study involving multiple laboratories [30]. This is critical given previous claims that the detection of unconjugated BPA in serum is merely a reflection of BPA contamination during either sample collection or assay [10]. Thus, the presence of unconjugated BPA in this carefully controlled analysis supports the contention that the presence of bioactive BPA in human tissues is real based on biomonitoring studies in which controls for contamination were included [3]. These conclusions are supported as well by our parallel experiments using ^3^H-BPA, demonstrating the presence of parent (non-conjugated) BPA in all fluids and extracts examined by radio-HPLC.

Adoption of the serum method and its validation in amniotic fluid facilitated the direct, simultaneous analysis of BPA and its two major metabolites in amniotic fluid. To our knowledge, our method is the first to achieve sensitivities below 1 ng/mL for all three analytes in amniotic fluid [35]. This allowed for an accurate and more sensitive measurement of BPA and its metabolites in this biological compartment, enabling better and longer tracking of these analytes in pharmacokinetic studies.

Our studies of pregnant rhesus females were carried out at two gestational stages, late second trimester (100 days gestation) and near term (150 days gestation). Our findings confirm that BPA delivered by gavage is readily transferred to the fetal compartment. The collection of matched maternal and fetal samples over a 4 hr period at two different stages of gestation allowed us to directly compare levels in maternal and fetal serum as well as amniotic fluid. Further, pharmacokinetic analysis based on the monitoring of unconjugated BPA, as well as BPA-G and BPA–S was validated by the results of a ^3^H-BPA based study run in parallel, since no other major metabolite could be detected in serum or amniotic fluid samples.

The levels of unconjugated and conjugated BPA in maternal serum in this study were predicted based on our previous study of orally dosed rhesus monkeys [7], and a daily dose of 400 μg/kg/day produced the expected levels of bioactive BPA in maternal serum. Importantly, the levels of unconjugated BPA in this study were similar to those measured in numerous human studies [32,36]. Thus, levels of BPA measured in fetal serum and amniotic fluid should be directly relevant to levels in pregnant women.

An important aspect of the current study is that, on days when sampling occurred, BPA was given via gavage to remove the influence of sublingual absorption that enhances the concentration of unconjugated BPA in serum [9]. Therefore, the data in Fig 1 show a much higher peak level of maternal BPA-G than in our previous study in which BPA was administered in dried fruit [7]. Gavage dosing maximizes first-pass metabolism in the liver, thus resulting in lower unconjugated and higher BPA-G levels than would be expected from any other route of exposure [6,9]. Further, although a rapid reduction in BPA-G in maternal blood over the 4 hr sampling period was evident at both 100 and 150 days gestation, measurable levels of conjugated BPA in the fetal compartment (serum and amniotic fluid) not only were present at 1 hr but persisted and even slightly increased over the 4 hr period. This suggests that fetal BPA exposure following a single oral administration persists far longer than previously thought, and is consistent with the slow rate of BPA-G clearance from fetal serum recently reported in sheep [27]. These data suggest that if, as predicted [2], BPA exposure for the US population is continuous and from multiple routes, fetuses would be expected to have higher levels of BPA for longer periods of time relative to their mothers.

Although levels of BPA-G and BPA-S were lower in fetal serum and amniotic fluid than in maternal serum, they did not show the rapid decrease that occurred in maternal serum. These results are similar to findings from studies in the pregnant ewe and mouse that suggest these BPA metabolites are trapped in the fetal compartment [24,35]. They differ, however, from our previous finding that levels of conjugated BPA in fetal serum and amniotic fluid were dramatically higher than in maternal serum on both 100 and 150 days gestation when pregnant monkeys were administered BPA chronically via subcutaneous Silastic implants, a method of administration chosen to more closely mimic chronic BPA exposure. We previously reported an inverse relationship between the concentration of unconjugated BPA and the ratio of conjugated:unconjugated BPA in maternal serum [7]. Routes of exposure that lead to a high ratio of conjugated:unconjugated BPA, such as intra-gastric gavage, will thus lead to lower concentrations of unconjugated BPA that can pass from maternal to fetal blood; transplacental transport of BPA-G from maternal to fetal compartments does not appear to occur [35]. Thus, although the current findings from rhesus monkeys are relevant to humans, they likely underestimate actual human fetal exposure, since much of human exposure is thought to be by non-oral routes that result in dramatically higher serum unconjugated BPA relative to the oral or intra-gastric routes [1–2,6].

In parallel with the pharmacokinetic study, our study of the metabolic fate of ^3^H-BPA administered by gavage to a non-pregnant and third trimester (140 days) pregnant female confirmed the rapid plasma peak (1 hr) of BPA residues in serum, and the persistence of detectable residues up to 24 hr post-dosing. The predominance of BPA-G in serum and presence of minor quantities of the sulfate conjugate and unconjugated BPA were confirmed and, as expected, BPA residues (metabolites and parent compound) were extensively excreted in urine, with very limited fecal excretion (below 0.2% of the administered dose). However, the persistence at 24 hr of significant amounts of radioactivity in the gastrointestinal tract (~2% of the administered dose) and in bile (similar quantities) provides strong evidence of enterohepatic cycling of BPA residues. This has been well described in nonpregnant animals in both rodents and primates [37–39]. Radio-HPLC profiling of bile extracts demonstrated the presence of BPA-G (major metabolite), parent BPA, BPA-S, as well as a bile-specific doubly conjugated metabolite (*ca*. 10% each). The three conjugates are expected to be cleaved back into the parent compound at the level of the intestinal tract, primarily by bacterial enzymes [40] and subsequently reabsorbed, contributing to the measurable levels of residues detected at 24 hr in tissues and in the fetal compartment.

At 24 hr, all tissues assessed contained measurable amounts of radioactivity, corresponding to 0.3% of the total administered dose. In adults, residual levels of ^3^H-BPA in tissues ranged from 10–100 ppt, with the exception of the liver and kidney (150–400 ppt). In addition, the adult uterus and ovaries (200–700 ppt) were obvious targets of BPA. Although prior experimental evidence suggested that the ovary is strongly targeted [16], our data provide the first clear evidence that BPA does in fact track to this tissue in nonhuman primates. Further, although BPA residues have previously been demonstrated to be present at 24 hr in the uterus and ovaries of exposed mice [24], in this species, the levels were lower than those measured in mouse liver.

This species difference in reproductive tissue vs. liver may reflect the higher levels of circulating estrogen in primates, including humans. For example, during gestation, levels in mice peak at around 60 pg/mL [41] compared to primate levels of 500 pg/mL or more [42] and potential differences in receptor density and affinity and membrane characteristics may also affect relative binding sites for BPA.

However, the differences between rodents and primates in maternal serum estradiol are complicated by the fact that rodent fetuses produce the estrogen-binding plasma protein alphafetoprotein at high levels, which results in a much lower (about 10-fold) percent free serum estradiol concentration (the bioactive fraction) in fetuses relative to adults [43]. In contrast, in rhesus monkeys and women, sex hormone binding globulin is produced in the maternal but not the fetal liver, so total serum estradiol concentration is higher in the primate mother relative to the fetus [44], which is the opposite of what occurs in rodents [45]. This results in a similar serum concentration of free (bioactive) estradiol in the rodent and primate fetus even though there is a marked difference in total serum estradiol.

The ^3^H-BPA study, also confirms that BPA reaches fetal tissues and fluids in rhesus monkeys in which adverse effects in female offspring due to maternal BPA exposure have been reported in experimental studies of the ovaries [18], uterus [20] mammary glands [19], and brain, heart and lung [21–23]. In the current study, ^3^H-BPA residues were present in all tissues and fluids in the single female monkey fetus examined, with concentrations ranging from 15 (fat) to 130 (lung) ppt. Furthermore, BPA residual levels in several tissues, including lung, brain, and heart, were comparable in the fetus and dam. Because BPA interacts with both nuclear estrogen receptors-α and –β as well as cell membrane–bound estrogen receptors [46], relatively high receptor levels in these tissues might account for the residual levels. Specifically, these tissues have been identified as having higher levels of estrogen receptor during fetal development [47–48].

## Conclusions

Monkeys in both the pharmacokinetic and metabolic fate study were administered oral BPA; either as a single dose or a daily dose over the course of the study. All animals showed measurable unconjugated and conjugated BPA in the fetal compartment and slow clearance compared to maternal serum. As discussed above, quickly swallowed BPA is most rapidly metabolized compared to other routes of exposure. Thus, the single oral dosing via nasogastric tube protocol used on the sample collections days in this study represents the scenario that would lead to the most rapid maternal metabolism and excretion and, presumably, the lowest level of fetal exposure. Multiple exposures per day would potentially result in higher fetal levels of BPA, and data from human studies suggest that BPA exposure is nearly constant and results from multiple routes of exposure [1–2]. Further, because non-oral BPA exposure leads to lower conjugated:unconjugated BPA ratios [7,11], it is likely these rhesus studies have less unconjugated BPA fetal exposure that what is experienced in humans.

This study demonstrates that BPA is readily transferred from maternal to fetal serum and amniotic fluid in primates after oral exposure. Furthermore, rapid processing and excretion of BPA residues, with a predominance of glucuronic acid conjugation and major urinary excretion, did not prevent substantial fetal exposure to unconjugated BPA. Importantly, the major BPA metabolites, BPA-G and BPA-S, were found in the fetal compartment and persisted even as maternal levels of these metabolites were declining. Lastly, the presence of small quantities of both the sulfate metabolite and unconjugated BPA in most tissues and fluids examined supports the hypothesis of enterohepatic cycling of BPA residues. Taken together, these data suggest that, in primates, rapid maternal processing of BPA does not alleviate the risk of exposure to the developing fetus, even after oral exposure that results in markedly lower serum levels of bioactive BPA relative to sublingual, transdermal or respiratory exposures. These findings elevate concerns about levels of current BPA human exposure from potentially a large number of unknown sources and the risks posed to developing fetuses.
